# Knowledge, attitudes, behaviors, and consequences of geogenic fluoride exposure on oral and dental health in Nakuru, Kenya

**DOI:** 10.3389/froh.2026.1775671

**Published:** 2026-03-20

**Authors:** Lavender Awino Okore, Rahul Aggarwal, Diana Ross Awuor, Patrick Mbullo Owuor

**Affiliations:** 1 Department of Psychology, Faculty of Health Sciences, Institute of Social Studies, University of Debrecen, Debrecen, Hungary; 2 University of Miami Leonard M. Miller School of Medicine, Miami, FL, United States; 3 Department of Youth Work and Community Development, Leeds Beckett University, Leeds, United Kingdom; 4 Department of Anthropology, Wayne State University, Detroit, MI, United States

**Keywords:** dental health, fluoride, fluorosis, oral health, water insecurity

## Abstract

**Background:**

Although efforts to improve household water security have significantly advanced progress toward Sustainable Development Goal (SDG) 6, environmental hazards, such as geogenic fluoride contamination, continue to threaten the lives of billions of people who rely on unimproved water sources. While awareness of the harmful effects of high fluoride levels in drinking water exists, research on knowledge, attitudes, and behaviors related to fluoride and fluorosis remains limited, particularly among households in areas with naturally elevated fluoride levels.

**Methods:**

We therefore examined knowledge, attitudes, and behaviors related to fluoride in drinking water among families living in Nakuru County, Kenya, where geogenic fluoride occurs naturally. We conducted semi-structured surveys with 52 individuals living in Nakuru County, Kenya, where water samples exceeded the WHO acceptable levels for drinking water.

**Results:**

Most participants (81%) demonstrated a good understanding of fluoride and fluorosis, including their presence in water and their effects on health, such as dental and skeletal fluorosis. However, despite this knowledge, some participants (67%) exhibited negative attitudes, showing little to no concern. While some participants (35%) reported correct practices, such as using alternative sources, a majority (65%) engaged in incorrect practices, such as boiling and chemically treating their water, or persistently brushing their teeth to remove dental fluorosis.

**Conclusion:**

Environmental hazards, such as fluoride, continue to challenge water security and pose heightened health risks, including oral health risks. In resource-limited settings, implementing interventions to shift attitudes and behaviors will be necessary to effectively translate existing knowledge.

## 1 Introduction

Household water insecurity, defined as the lack of accessible, stable, affordable, and safe water for household use (1), is critical for physical, nutritional, and psychosocial health (2–4). It is linked both directly and indirectly to several Sustainable Development Goals, such as No Poverty (SDG 1), Zero Hunger (SDG 2), Good Health and Well-being (SDG 3), and Clean Water and Sanitation (SDG 6), among others (5). Although many nations strive to achieve these goals, SDG 6 remains elusive, particularly in sub-Saharan Africa, where climate change, population growth, and inadequate infrastructure present significant challenges (6). In Kenya, for example, an estimated 9.9 million people—almost 20% of the population—rely on unimproved and often contaminated water sources for their daily water needs (Njoroge et al., 2024), with major concerns including water quality issues such as bacterial infections (E. coli) and chemical pollutants (fluoride and lead), as well as water availability and affordability-related concerns (7–10).

While research on household water insecurity has emphasized issues related to water availability, accessibility, and use (1, 11, 12), in low-resource settings, especially in sub-Saharan Africa, the focus has been on physical access and affordability (13, 14). Although this emphasis has been vital in delivering reliable water sources to thousands of communities, it also means that concerns about water quality, particularly those caused by environmental contaminants, have not always received the attention they deserve. For example, in the fluoride-endemic Rift Valley region of Kenya, water intervention efforts consistently target groundwater sources with boreholes and shallow wells, despite existing knowledge of fluoride contamination (15, 16).

Environmental pollutants, such as fluoride, however, pose a challenge to household water security (17, 18). The World Health Organization sets the recommended limit for fluoride in drinking water at 1.5 ppm (19). Although small amounts of fluoride in drinking water promote oral health, naturally occurring fluoride at higher levels can present significant challenges for household water needs (20). In regions with naturally occurring fluoride, groundwater use may increase exposure risk due to mineral weathering and leaching from rocks and sediments (21). Fluoride ions in drinking water bond with calcium in teeth and bones, leading to their degradation and dental and skeletal fluorosis (17, 20, 22). Dental fluorosis or mottled enamel is the staining of teeth characterized by damaged or corroded surfaces due to fluoride exposure, mainly in the early stages of tooth development (23). In India, for example, a study on the prevalence of dental fluorosis among adolescents in schools of Greater Noida, Uttar Pradesh, revealed that over 70% of young adults suffered from fluorosis due to drinking contaminated water. Similarly, skeletal fluorosis resulting from excess minerals in bones has been reported in populations with excessive fluoride exposure through drinking water, leading to bone deformities and debilitating diseases (24). In a study conducted in the Kerio Valley, higher fluoride levels in water exceeded acceptable limits, and residents reported both dental and skeletal fluorosis (25).

Despite limited data due to a lack of research, oral health problems remain a primary concern in Kenya. The 2022 National Oral Health Policy report states that over 98% of adults have periodontal or gum disease, with significant cases of oral cancer and pre-cancerous lesions. Among children, approximately 40% have dental caries, more than 90% have periodontal disease, and 41% have dental fluorosis (26). The policy acknowledges that dental diseases often stem from modifiable risk behaviors like eating foods high in refined sugars, practicing harmful traditional habits, and using tobacco and alcohol. However, it does not consider the significant impact of environmental pollutants, such as fluoride, or resource insecurity issues, such as water scarcity, as possible contributing factors. Despite potential concerns about fluorosis in oral health, perceptions, attitudes, and beliefs about fluoride remain largely underexplored. Therefore, using the Knowledge, Attitudes, and Behaviors (KAB) framework, this study aimed to qualitatively explore knowledge, attitudes, and behaviors regarding fluoride and fluorosis, and how these may influence oral health in Nakuru, Kenya.

### 1.1 Knowledge, attitudes, and behaviors (KAB) framework

The Knowledge, Attitudes, and Behaviors (KAB) framework is a useful model for health promotion and behavior change (27) that offers insights into why individuals behave the way they do (28). The KAB framework shows that when people gain knowledge—such as information and skills—they often experience changes in attitudes, such as increased motivation and clearer intentions (29). These positive shifts then encourage them to try new behaviors, making this model a guiding tool for understanding how learning can lead to meaningful action. The KAB framework suggests that our behavior results from our attitudes, which are shaped by knowledge (29). While knowledge here primarily refers to factual information, beliefs, values, and norms are central to interpreting, utilizing, and maintaining fidelity to that knowledge (30). Although there is abundant literature on knowledge, attitudes, and behaviors toward fluoride (18, 27, 31), these studies have tended to focus on the value of educating the public about the benefits of water fluoridation for oral health, especially stronger teeth. Moreover, these studies have been conducted in high-income countries such as the United States, where water fluoridation is a standard practice (31, 32). However, there has been minimal attention to rural communities where fluoride occurs naturally, infrastructure is poor, and access to education and safe drinking water is limited. In these settings, knowledge, attitude, and behavior toward fluoride have been lacking.

In oral health, the KAB framework demonstrates that having knowledge about oral health in resource-limited settings doesn't always result in healthy behaviors. For instance, a study of 12-year-old schoolchildren in Iran found that although they had good knowledge and positive attitudes towards oral health, this did not translate into better oral health practices. Rural students, in particular, had poorer outcomes compared to those in urban areas (33). Similarly, a study in Romania, conducted among 258 patients in rural areas, assessed self-perceptions of oral health, knowledge, and attitudes toward oral health, dental hygiene practices, and eating habits. The findings showed that while basic knowledge of oral health among rural patients is satisfactory, their practices do not reflect this knowledge (34). In Kenya, a study among schoolchildren revealed that less than half understood what causes tooth decay or how to prevent it. Also, only about half of the students brushed their teeth twice a day (35). While these studies highlight the importance of knowledge, attitudes, and behaviors related to oral health, examining the KAB framework within communities with naturally occurring fluoride—where knowledge reflects community values, beliefs, and norms—would be helpful.

## 2 Methods

### 2.1 Study area

This study was conducted in four villages: Kelelwet, Kigonor, Parkview, and Kipsibol Sublocations, in Barut Sub-County, Nakuru County. Located in the Rift Valley, Nakuru County has relatively high geogenic fluoride levels, posing significant health concerns. For instance, a 2018 study found that 87.5% of the boreholes surveyed exceeded the WHO guideline of 1.5 ppm for dissolved fluoride (20). Similarly, in a smaller study of 52 drinking water samples from Nakuru, 56% had elevated fluoride levels, most of which were sourced from community boreholes, shallow wells, and rivers (36).

Although classified as peri-urban with Nakuru Town West, Barut Subcounty is mainly rural, with subsistence farming as its main occupation. The area has inadequate infrastructure, including inaccessible roads, limited electricity, poor sanitation, and little to no piped water infrastructure. While some families have access to boreholes and rainwater collection systems for drinking water, most households rely on unimproved water sources, such as rivers, for domestic use. Despite being perceived as a safe water source, most boreholes are located outside homes, are untreated, and contain high levels of fluoride (37).

### 2.2 Study design

This was a sub-study of a mixed-methods, cross-sectional proof-of-concept study evaluating the accuracy and usability of a rapid, at-home fluoride test for drinking water in Nakuru, Kenya. In the proof-of-concept, households were instructed on how to test water using a point-of-use biosensor and then asked to test water samples from their homes. They were then asked to interpret the results by comparing the color changes; yellow indicated elevated fluoride. Research assistants confirmed whether their interpretations were correct or incorrect. Results were compared to an established fluoride measurement method using an iron-sensing electrode (36). After the results were shared, participants were asked about their experiences with fluoride and their knowledge, attitudes, and behaviors (KAB) regarding fluoride. Findings from the proof-of-concept revealed that although most residents were aware of fluoride, knowledge on treatment and prevention was lacking (36). In this sub-study, we aimed to better understand the reasons for the lack of knowledge about treatment and prevention, and whether knowledge of fluoride influenced people to adopt better practices. To do this, we re-examined the qualitative responses and field notes gathered during our fieldwork.

### 2.3 Recruitment and screening methods

While the recruitment strategy and sampling procedure have been reported elsewhere (36), in brief, participants were purposively sampled and included in the study if they were a) aged 18 and above b) living within the specified region for more than 3 months and used water from community boreholes and other shallow wells, c) living with a child/child under the age of 10 years, d) willing to share information regarding their water sources, and e) were familiar with household water acquisition and use. The sample size of 52 participants was determined based on the minimum required to establish test accuracy. We worked with community leaders in all community activities. First, we met with community leaders and organized community sensitization meetings before collectively identifying the most frequently used community boreholes in Barut ward, Nakuru County. In total, six boreholes in six sublocations were selected. The research assistants, alongside a community mobilizer, then approached households near the boreholes and used them as their primary water source. A total of 10–12 participants were recruited from each site.

### 2.4 Ethical considerations

This study received ethics approval from the Amref Ethics and Scientific Research Committee (ESRC P1003/2021) and the Northwestern University Institutional Review Board's Committee on Human Research (STU00215306). Before data collection, all participants provided written informed consent. Participants received an honorarium of KES 1,000 (approximately USD 8).

### 2.5 Data collection

Data for this sub-study were drawn from the survey's qualitative components, including field notes and conversations that were initially not part of the survey. In brief, two research assistants conducted the surveys, which were programmed into Open Data Kit (ODK); interviews took 20–30 min. The survey included both closed- and open-ended questions about experiences with water insecurity and knowledge, attitudes, and behaviors (KAB) related to fluoride, including its perceived health effects.

In this study, we defined knowledge as the ability to accurately identify sources of fluoride and associate fluorosis with excessive fluoride exposure. We also described attitude as socio-cultural perceptions about fluoride and its effects. Finally, we defined behavior as household and individual practices related to the prevention and treatment of fluoride exposure.

The KAB questions were developed based on our prior experiences and community members' feedback before the research began. Some of the questions included: What is your understanding of fluoride? What is your knowledge of fluorosis? What are the effects of fluoride exposure? How can you prevent fluorosis? How can you treat fluorosis? We also asked about the precautions taken to avoid fluorosis (Supplementay Material S1).

### 2.6 Data analysis

Demographic data was exported from ODK to Excel. Open-ended questions were extracted and exported to a Word document, which was subsequently imported into Atlas.TI for qualitative content analysis (QCA). The QCA is a systematic method that emphasizes counting the frequency of specific words, themes, or concepts in a text (38).

Using Microsoft Excel, the second author identified keywords and generated a frequency count of how often participants mentioned each keyword within each prompt category. The prompt categories were organized into knowledge, attitude, and behavior to develop a coding schema. All authors assessed the coding schema and their descriptions. After determining response frequencies under these categories, the first and second authors linked them to participant experiences during the first round of coding. Afterwards, all authors conducted a rigorous content analysis and assessed the initial coding to ensure consistency while exploring patterns and emerging themes.

## 3 Results

### 3.1 Demographics

Detailed demographic characteristics have been published elsewhere (36). Briefly, the median age of the participants was 41, with an interquartile range of 32–50. 73% were women, and only 38% had completed secondary education. Virtually all participants (87%) were engaged in informal employment as a source of livelihood (Table 1).

**Table 1:** Socio-demographic characteristics of participants in the Kenya fluoride study.

Sociodemographic Characteristics	Participants (*N* = 52)
Age (years)	
Median (IQR)	41 (32, 50)
Sex, *n* (%)	
Female	38 (73.1)
Male	14 (26.9)
Education, *n* (%)	
None	3 (5.8)
Some Primary	11 (21.2)
Completed Primary	10 (19.2)
Some Secondary	8 (15.4)
Completed Secondary	8 (15.4)
College/University	12 (23.1)
Employment, *n* (%)	
Farming	15 (29.5)
Small Business	21 (40.4)
Unemployed	10 (19.2)
Other	6 (11.5)
Water source, *n* (%)	
Borehole/well	38 (73)
Rainwater	11 (21)
others	3 (5.8)
Household Size	
Mean (SD)	4.9 (1.8)

To better understand perceived knowledge, attitudes, and behaviors, we analyzed open-ended responses and identified key themes under each domain, as illustrated below (Table 2).

**Table 2:** Themes associated with KAB in open-ended interviews among residents of barut, nakuru, Kenya.

Domain	Theme	Characterization
Knowledge: Participants understanding of fluoride, its exposure and consequences	Defining fluoride and fluorosis	Fluoride is foreign salt-like substance found in water
	Sources of fluoride	Fluoride is found in water and food we eat
	Consequences of fluorosis	Fluoride has health consequences such as dental and skeletal fluorosis
Attitude: Participants perception including reaction to fluorosis effects	Stigma	Teenagers with dental fluorosis feel shy and uncomfortable
	Less worry	Fluoride was here before us and our parents survived it
	Less action	We have no solution; nothing works not matter how we try
Behavior: Participants practices preventing fluorosis	Boiling of water	We mostly boil water to remove the acidity
	Chemical treatment	We also use chlorine to treat water
	Switching water sources	Sometimes we use rainwater or bottled water or find alternative borehole
	Brushing teeth	We are told to brush our teeth so we continue, and hope things will change
	Other practices	We are also told to drink a lot of milk to reduce the effects of fluoride

### 3.2 Knowledge on fluoride and fluorosis

**Figure 1:**
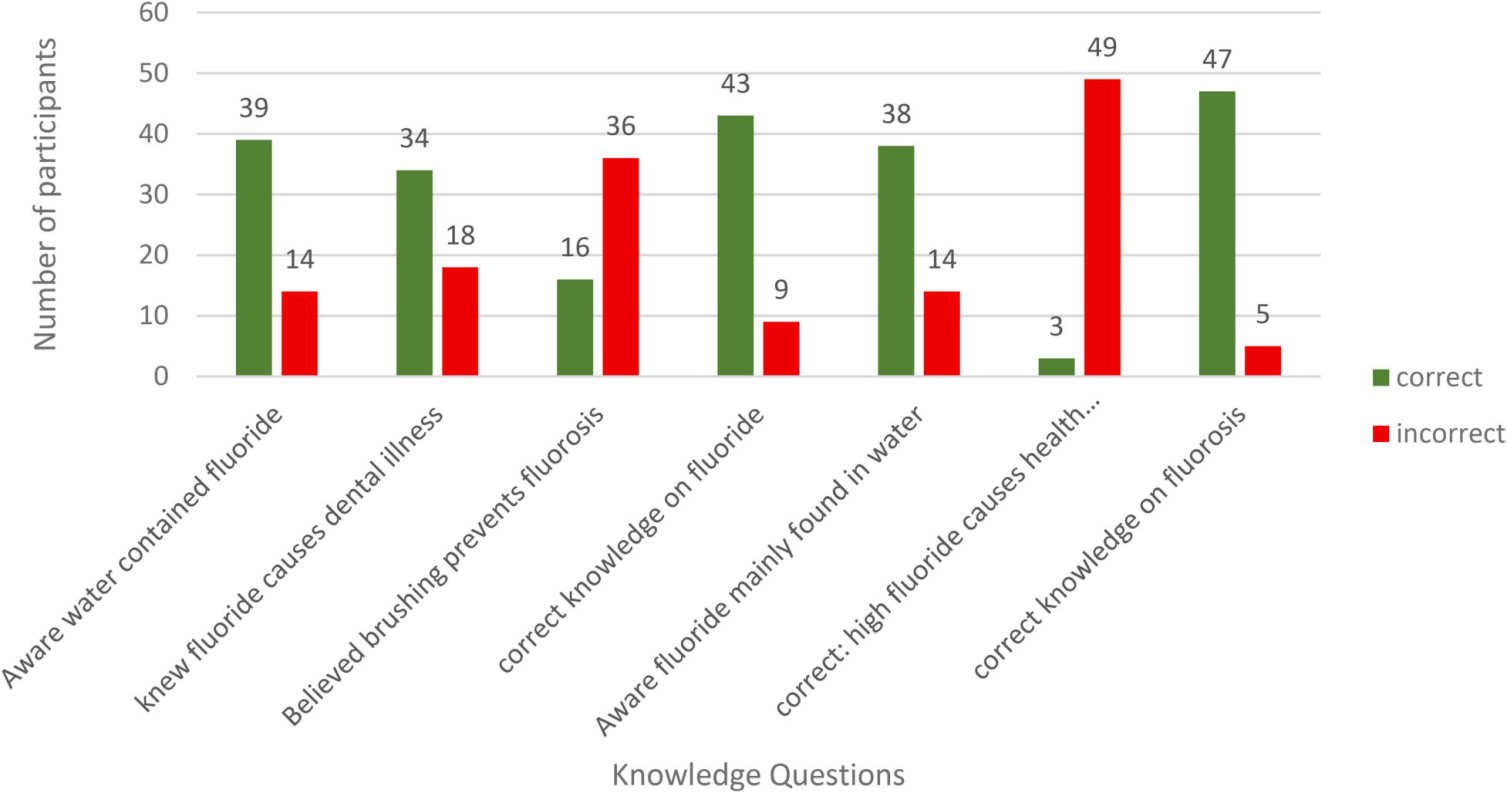
Knowledge of fluoride and fluorosis in barut sub-county, nakuru, Kenya (*N* = 52).

In our survey data (*n* = 52), respondents demonstrated strong awareness of fluoride and fluorosis. Overall, 42 (81%) respondents showed correct knowledge of fluoride. An accurate understanding included knowing that fluoride is mainly found in water. Most health effects resulted from drinking excessive amounts of fluoride-contaminated water. Similarly, there was a higher level of knowledge about fluorosis, with 47 (90%) of respondents demonstrating correct knowledge, including dental and bone-related consequences, as well as a broader understanding of the disease caused by excess fluoride exposure (Figure 1).

In the qualitative data, 59% of participants reported daily struggles with dental issues. Some (12%) reported corrosion or chipping of their teeth or those of family members. They believed this was caused by foreign “acid” in the water. For these participants, fluoride was described as an “acid” or water with acidic properties that harm teeth and bones.

We have four children, but if you look at their teeth, you don't see anything because they keep falling off and chipping away as they grow. (40-year-old woman, PID 1−02)

However, participants who reported dental problems also reported experiencing pain and difficulty cleaning their teeth, chewing, or eating certain foods.

Our kids cannot eat things such as meat that require chewing because their teeth are broken and have grown weaker with time. (42-year-old woman, PID 1−03)

About 34% of participants reported observing the effect of fluoride in their children early in life. These participants understood the pattern of skeletal and dental issues arising from fluorosis as one narrated:

I remember with each child, I waited for the baby to grow and kept hoping that the new ones would be stronger and endure. But that was not the case. At the age of 10, they began losing other teeth, which were also discolored. (27-year-old woman, PID 1−04)

### 3.3 Attitudes toward fluoride and fluorosis

**Figure 2:**
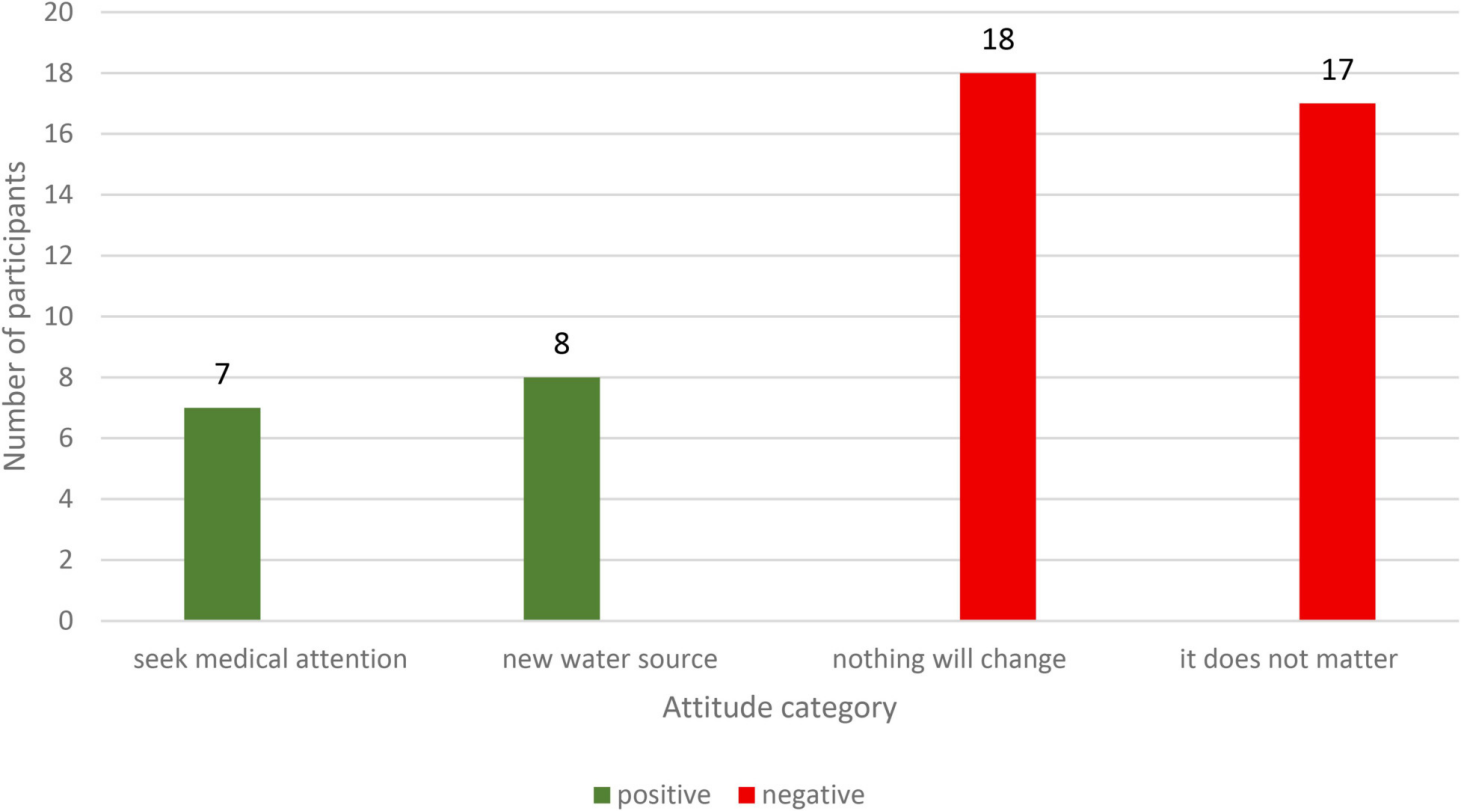
Attitude toward fluoride and fluorosis in barut sub-county, nakuru, Kenya (*N* = 52).

In the survey, we asked participants if they were worried about their water sources or fluoride levels, and whether they would take any action. A majority, 35 (67%), said they were less concerned about fluoride because they believed things would stay the same and therefore it didn't matter to them (Figure 2).

However, some participants (37%) reported that young people often felt ashamed of their discolored teeth and avoided gatherings out of fear of being ridiculed by peers.

Our eldest, now 16, struggles to communicate with people. She speaks less in public and does not participate in community activities. She often keeps to herself, referring to the negative comments she receives from her peers about her teeth. Her school performance has drastically deteriorated. (40-year-old woman, PID 1−02).

It's a huge issue for young people. My 21-year-old son, who's in college, still wants me to “clean” his teeth for him, even though I haven't done it. Also, I tried cleaning my 18-year-old daughter's teeth for her, but it still came back. They hardly ever talk in public or participate in social activities with their friends. (54-year-old woman, PID 1–12).

### 3.4 Households' behavior to prevent excessive fluoride exposure

**Figure 3:**
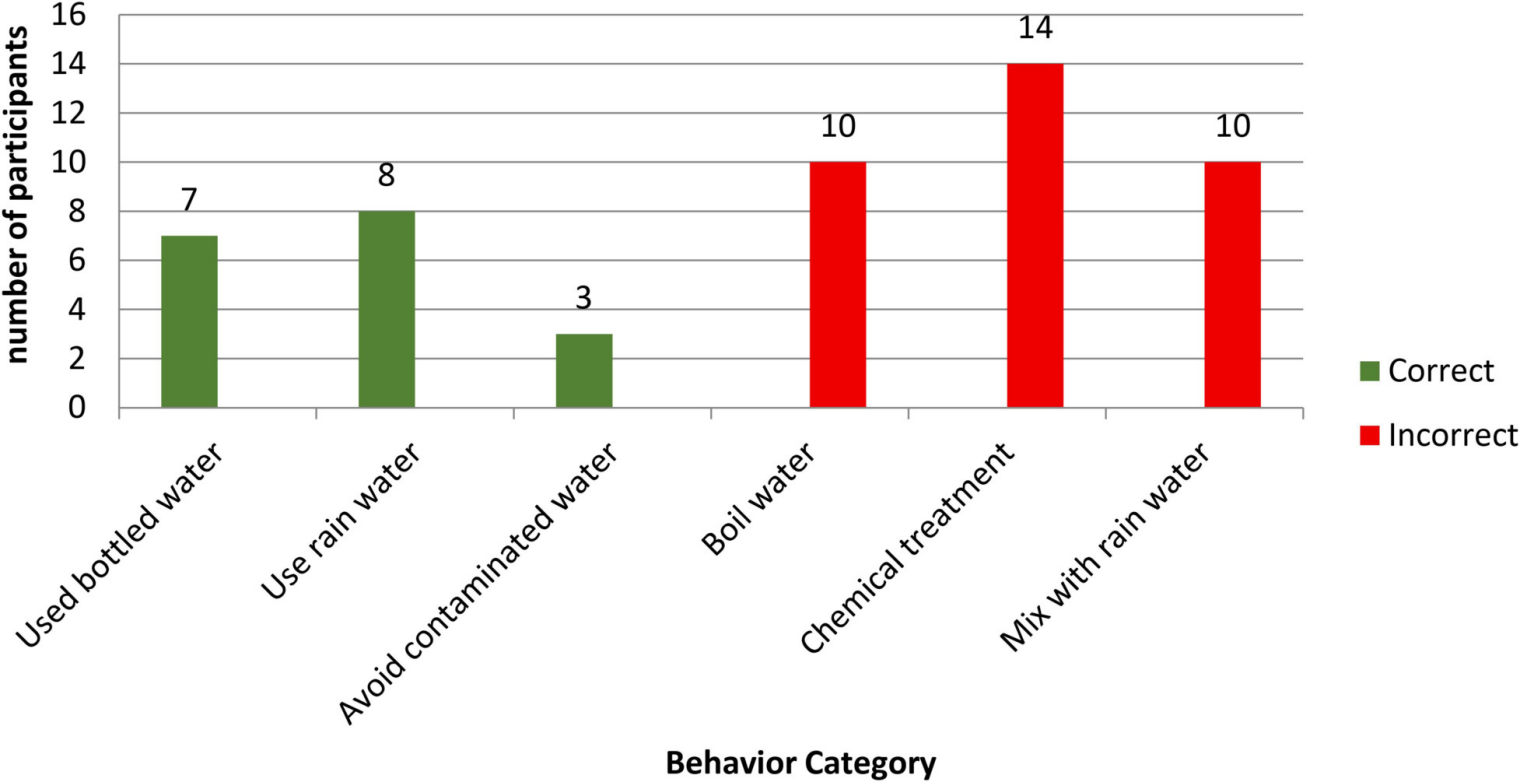
Behavior toward fluoride and fluorosis in barut sub-county, nakuru, Kenya (*N* = 52).

Regarding fluoride use and fluorosis, only 35% (18) of participants reported proper fluoride management practices, whereas 65% described incorrect behaviors like boiling water and chemical treatment (Figure 3).

In the qualitative data, all participants reported that they had never found any successful remedy or cure. Participants reported that multiple generations of their families had lived in the same area with fluoride exposure for many years, and that community efforts to address the issue had not been successful. They described both preventive and treatment practices, such as using alternative water sources, boiling water, brushing teeth, and drinking milk. We discuss some of these practices below.

#### 3.4.1 Preventive practices

##### 3.4.1.1 Alternative sources of water: source switching

Although there was no clear intervention to lower fluoride levels in community water sources, many participants (33%) reported that families were turning to alternative preventive measures, such as choosing other water sources. The most common alternative was rainwater (22%), which was kept solely for drinking. Others mentioned that they would mix water from various sources, particularly rainwater or bottled water, and borehole water, to enhance the taste and hopefully decrease fluoride levels.

I collect rainwater for drinking, but during dry months, we rely on storage containers to store it when it does rain. Here, rainwater is prized, and having a rain gutter to harvest it is a sign of wealth. There are seasons when we buy rainwater from our neighbors. It is cheaper than bottled water (3-year-old woman, PID 1–08)

Other participants (16%) reported asking their children and family to use either bottled or rainwater when brushing their teeth. However, accessing bottled water proved challenging due to its cost. Additionally, other people reported that households unable to afford it would mix rainwater with drinking water or rely entirely on rainwater for cooking and drinking.

Sometimes, I mix rainwater with borehole water to diminish the saltiness. The salty borehole water in this area has caused my children's teeth to become so brown that it disgusts me. Unfortunately, we know this problem stems from the water, yet there is nothing we can do about it. Household (41-year-old woman, PID 1–06).

In response to the widespread presence of fluoride in the Nakuru region, households utilize multiple sources for various purposes, including river water for laundry, bottled water for drinking, and borehole water for cooking. This approach, which we refer to here as “source switching,” was perceived by participants as a means of reducing or avoiding the consumption of contaminated water. Most participants said they understood fluoride as a type of salt in water. Therefore, they could tell the water was contaminated when it tasted salty, using their taste buds to detect fluoride.

We get our water from two different community boreholes. Our drinking water comes from a treated borehole, while water for other uses is fetched from a non-treated borehole or the river down there. We also store them differently. The untreated water is stored in small plastic containers in the house for use (especially for cleaning). All of these sources contain salt, so we taste them first to determine the level of salt and decide how to use them. The treated water has little salt, so we prefer it for cooking, while rainwater has no salty taste, so we use it for drinking. (37-year-old man, PID 1−01).

##### 3.4.1.2 Milk or water for fluoride

Some participants (2%) also reported encouraging children to drink a lot of milk rather than water, as they perceived this would reduce fluoride exposure.

We consume a substantial amount of milk. If you don't own a cow, you can purchase milk from neighbors or the market. We also encourage our children to drink milk instead of water. As you can see, there's a jug of milk over there that they can access at any time. I can say this has helped many people reduce the effects of this salty water. (42-year-old woman, PID 1–11).

#### 3.4.2 Treatment practices

##### 3.4.2.1 Water boiling

To reduce fluoride levels, some participants (5%) reported that they generally preferred boiling their water for an extended period. However, it was also challenging during wet seasons when they were forced to use charcoal for cooking.

We try different strategies to reduce the amount of salt in the water. For example, my family mixes borehole water with rainwater before boiling it. After boiling the water, we let it cool and store it for drinking. The water tastes better when we do this (37-year-old woman, PID 1–18).

##### 3.4.2.2 Chemical treatment

Some participants (7%) reported using chemical methods such as chlorination to treat their water at the point of use. However, participants indicated that chlorine-based treatment required additional funds to maintain; hence, it was done only occasionally when one could afford it.

There are instances when I use chlorine for water treatment, especially when the water appears extremely dirty. I buy from the nearby shop, but it is expensive, and I cannot afford to use it regularly. It is better than boiling because it not only improves the taste but also makes the water clearer. (41-year-old woman, PID 1–13).

By using chemicals like bone char and using alternative water to reduce it. There are some tablets we used to be given to remove fluoride, but they only reduce it, not eradicate it. Additionally, some tablets were provided to remove fluoride, but they only reduce it, not eradicate it. (51-year-old man, PID 1–14).

## 4 Discussion

In this study, which investigated the knowledge, attitudes, and beliefs of households residing in an area affected by geogenic fluoride in Nakuru County, we found that most households had correct information about the causes of fluoride and fluorosis. However, this high level of knowledge did not lead to effective prevention or treatment practices. Consequently, families adopted ineffective practices, such as boiling water, blending borehole water with rainwater, chemical water treatment with chlorine-based tablets, and persistent tooth brushing, to treat dental fluorosis. Similarly, the high level of knowledge did not translate into positive attitudes toward fluoride's health effects. Most participants indicated they would do nothing since that was the norm. Additionally, they reported that they expect things to remain the same in the future.

While most participants showed little concern and a poor attitude, some said they were worried about their dental health and felt stigmatized because of discoloration and tooth damage. For these participants, dental fluorosis was seen as a hindrance to integration into community social activities. For example, young people with discolored or corroded teeth felt shy and discouraged from taking part in community events and social activities. While stigma arising from household water insecurity has been attributed to the shame of borrowing water, not being able to shower, bathe, or do laundry (9, 39, 40), there is limited literature on stigma stemming from discolored teeth due to fluoride exposure. These results show the psychological effects of oral health and align with similar findings that emphasize integrating oral health with public health and noncommunicable diseases to improve oral healthcare utilization (26, 41).

Although excessive fluoride is well-established as deleterious (23–25), our research suggests that knowledge, particularly regarding the prevention of dental fluorosis, remains limited in these communities, hindering the adoption of practical strategies and health interventions. While traditional norms and lower health literacy have been reported to contribute to poor oral health in Kenya (26), our research suggests that greater knowledge of the health risks of excessive fluoride exposure may not necessarily lead to action or positive behavior change. Though these findings contradict the literature, which has found that access to accurate, trusted information can be crucial for driving behavior change in public health efforts (42, 43), they align with other research highlighting the disconnect between knowledge and action and emphasizing the importance of addressing individual social and environmental factors (44). In our view, the absence of practical solutions despite the community's efforts, along with minimal government support, served as a source of demotivation, underscoring the intention-behavior gap. Our study found that, despite years of fluoride exposure, participants lacked motivation to adopt effective prevention and treatment measures. We suggest that this lack of interest or motivation was due to years of inadequate public health interventions in this area.

In resource-poor settings, structural barriers limit the ability to overcome vulnerability (45). In this study, we found that limited household resources were a major driver of the knowledge gap, poor attitudes, and inconsistent practices. Participants recounted experiences detailing the lack of community infrastructure to treat fluoride, the absence of government support, and insufficient resources to manage fluoride as reasons for not taking the appropriate action. Naturally occurring fluoride can be difficult to manage, including its detection and treatment (18). Specialized tests are needed to measure fluoride levels, which can vary by location (46). Additionally, many fluoride detection methods rely on labs and require technical skills that households usually don't have, making them inaccessible to rural and low-income families (36). While there are promising efforts to develop point-of-use fluoride tests (36, 47) and build boreholes with fluoride treatment systems (48, 49), these technologies are expensive and not widely accessible to many resource-limited communities.

We also found that misinformation about fluoride treatment, such as brushing teeth to prevent or remove fluoride, was widespread and accepted among individuals. The lack of trusted sources of information, combined with minimal public efforts on fluoride education or mitigation, may have contributed to the information gap. Studies have shown that without trusted sources of information, people may rely on what they see around them, further solidifying their beliefs in those sources, regardless of their accuracy (50). Misinformation can lead to public health issues, as seen during the COVID-19 pandemic when people turned to traditional methods to treat or prevent the disease (51). Research on misinformation about fluoride highlights the critical role of social connections and experts in fluoride education, showing that inconsistencies prevent individuals from taking action (52). Though most misinformation on fluoride has focused on sources and effects of fluoride, our study reveals that misinformation about fluoride removal and treatment of fluorosis is rampant in resource-poor settings.

Although our study showed that families switched water sources to lower fluoride intake, we found no link between source switching and dental fluorosis. While evidence suggests that source switching is one of the most common strategies for managing water quality (53, 54), our study found that, for some households, the cost of alternative sources was also prohibitive. Our findings extend this work and reveal that families are adopting desperate strategies, such as switching water sources, to reduce fluoride exposure, which further increases their risk of exposure to other contaminants. Inadequate water infrastructure can complicate household water supply. Rural communities, especially in LMC, are often marginalized and do not receive adequate government interventions, experience poor infrastructure and basic services such as water and sanitation facilities (55). Evidence suggests that a lack of or inadequate water infrastructure may lead to health consequences due to increased reliance on unimproved sources (56). Consequently, these sources pose potential health risks, including concerns about water quality (57, 58).

Our study highlights challenges in children's food intake due to dental and skeletal fluorosis, including difficulties chewing certain foods. Though children's susceptibility to fluorosis, along with its lifelong effects, underscores the danger posed by fluoride contamination of drinking water sources during early years (25, 59), there is limited literature on how these effects may influence nutritional health. Our study identifies potential pathways by which household water fluoride exposure may compromise nutritional health. While the link between water insecurity and food is well established, this body of work primarily focuses on issues around availability, access, quality, and affordability. The effects of water pollution, such as fluoride, on dentition and food intake are lacking in existing literature. Our study expands on the link between water insecurity and food by highlighting the consequences of fluoride on dentition, thus contributing to a vast body of literature on the dietary effects of household water insecurity (2).

## 5 Study limitations

Our study had notable strengths, including a collaborative approach to recruiting participants and working closely with local administrators, community leaders, and mobilizers to engage the community and raise awareness of the project's goals. Additionally, by including all major community water points in the participant selection process, we ensured fair representation of various community perspectives. Despite these strengths, our study also faced limitations. For instance, although our research assistants were Kenyans, they were from outside the community and not fluent in the local language, so we used translation when participants did not speak English or Swahili. Secondly, because the parent study included household water testing for fluoride, which took nearly 3 h, many participants probably felt fatigued during data collection. Our research team's presence may have created power imbalances and potentially limited open interactions with participants. Additionally, due to the limited number of responses and the narrow scope of qualitative methods used, our data cannot be broadly generalized and may not capture the full diversity of community perspectives, which are often better reflected through methods like focus group discussions.

## 6 Policy implications

Oral health is a critical component of well-being. Poor oral health can affect psychosocial, nutritional, and physical health. Although knowledge, attitudes, and behaviors are crucial for improving oral health, their effectiveness depends heavily on available resources, particularly in contexts involving environmental factors such as fluoride. For practitioners and policy experts alike, promoting oral health knowledge must be accompanied by better access to essential resources to achieve positive outcomes. For example, while accessible point-of-use fluoride testing at the household level is a first step to information, communities in areas with naturally occurring fluoride are unlikely to change their water sources if no alternatives exist. Lastly, while knowledge can be instrumental in changing individual attitudes and behaviors, achieving sustainable community-wide change requires collective efforts. Therefore, practitioners should work closely with communities to strengthen advocacy for resources to effectively manage fluoride and its effects.

## 7 Conclusion

Oral health remains a concern, especially in resource-limited areas. Research on the link between water insecurity, fluoride contamination, and dental health should emphasize knowledge, attitudes, and behaviors to improve oral healthcare utilization. Although developing trustworthy sources of information about fluoride and its health effects is a step toward improving accurate knowledge, the psychosocial effects of fluorosis, such as stigma, may require multisectoral approaches, including integrating oral health into noncommunicable and public health sectors. Finally, attention should be given not only to improving water infrastructure but also to promoting better behavioral practices, especially those related to fluoride removal or fluorosis treatment.

## Data Availability

The original contributions presented in the study are included in the article/Supplementary Material, further inquiries can be directed to the corresponding author.
