# Supplementary material for: Knowledge, attitudes, behaviors, and consequences of geogenic fluoride exposure on oral and dental health in Nakuru, Kenya
Source: Front Oral Health. 2026 Mar 20;7:1775671. doi: 10.3389/froh.2026.1775671 (PMC13047096; doi:10.3389/froh.2026.1775671)
Supplement: Supplementary file 1 [file Table1.docx]

**Supplemental 1: Fluoride knowledge, attitudes, and behavior**

| 1. **Fluoride knowledge, attitudes, and behavior** |
| --- |
| I am now going to talk to you about your knowledge of fluoride and fluorosis. First, what is your understanding of [Fluoride]? |
| What is your understanding of [fluorosis]? |
| What are the effects of [Fluoride] exposure? |
| How can you prevent [fluorosis]? |
| How can you treat [fluorosis]? |
| How often do you worry about [fluorosis]? |
| Have you ever taken precautions against [fluorosis]? |
| Have you ever taken precautions against fluorosis? If yes, what were they? |
| Do you know of anyone who has been harmed by [fluoride] in water? Please tell me about the fluorosis—the person’s age, gender, how it affected their life, and if they got any treatment. Do they know where the fluoride was coming from? |
| What will you do differently now that you know about the fluoride levels in your water? |
